# A Japanese Patient With Progressive Word Deafness and Logoclonia

**DOI:** 10.7759/cureus.70045

**Published:** 2024-09-23

**Authors:** Atsuko Hayashi, Yasuji Yamamoto, Minoru Matsuda

**Affiliations:** 1 Department of Rehabilitation Science, Kobe University Graduate School of Health Sciences, Kobe, JPN; 2 Department of Biosignal Pathophysiology, Kobe University Graduate School of Medicine, Kobe, JPN; 3 Department of Neurology, Seizankai Group, Izuminomori Clinic, Sendai, JPN

**Keywords:** apraxia of speech, dat scan, logoclonia, non-fluent primary progressive aphasia, word deafness

## Abstract

Primary progressive aphasia (PPA) is classified into several types. However, syndromes that do not belong to these typical types of PPA have been reported, such as cases where only progressive apraxia of speech (AOS) is present in the early stages of the disease. Moreover, there have been a few case reports of patients with progressive word deafness complicated with logoclonia as a speech disorder. Herein, we report a case of degenerative disease with phonological paraphasia, AOS, impaired speech sound cognition, and logoclonia with disease progression. The written language comprehension of the patient was superior to that of spoken language, and she could communicate in writing. She was neurologically normal; had no apraxia, agnosia, or memory impairment; and was independent in activities of daily living. Although she had moderate hearing loss, her speech-related hearing loss was disproportionately high. In analyses of brain imaging, dilation of the left Sylvian fissure, atrophy, and hypoperfusion of the superior temporal gyrus were prominent, and hypoperfusion of the left lateral frontal lobe was observed. Cerebrospinal fluid examination suggested that Alzheimer’s disease (AD) pathology was unlikely, and decreased uptake was observed on a dopamine-active transporter scan. Word deafness was thought to be the most likely cause of the dissociation between spoken and written language in our patient. Our case and previous studies suggested that there is a syndrome presenting word deafness and speech movement disorders among patients with progressive communication disorders as a different entity from PPA. Although patients with this syndrome may experience agrammatism, inner language disorders are generally not as severe, and disorders in areas closer to the primary area, such as the motor and auditory areas, as well as cortical and subcortical disorders, are involved in this syndrome.

## Introduction

Primary progressive aphasia (PPA) is classified into the following three types: the non-fluent/agrammatic variant (nfvPPA), the semantic variant (svPPA), and logopenic variant PPA (lvPPA) [1]. However, syndromes that do not belong to these three types of PPA have been reported, such as cases where only progressive speech apraxia is present in the early stages of the disease [2-4]. Moreover, there have been reports of patients with progressive word deafness with apraxia of speech (AOS) [5-7], foreign accent syndrome (FAS) [8,9], or logoclonia [10,11]. Cases of the three types of PPA, along with progressive word deafness, have also been reported (nfvPPA [12], svPPA [13], and lvPPA [14]).

Here, we present a case with marked phonological paraphasia and AOS in the early stages of the disease, during which time the patient showed prominent auditory verbal comprehension disorders owing to progressive word deafness. There have been a few case reports of patients with progressive word deafness complicated with logoclonia as a speech disorder, and limited studies exist examining the successive neuropsychological assessment and detailed auditory tests, as well as neuro fluid tests and detailed neuroimaging investigations. Furthermore, the mechanisms underlying speech sound cognition and speech disorders with the disease progression remain unclear. Therefore, we described the symptoms and investigated the relationship between word deafness and speech disorders.

## Case presentation

The patient was a 74-year-old, right-handed female with 12 years of education. She complained that she had difficulty finding words and had not been able to communicate via telephone since the age of 68 years. The patient ran a restaurant with her husband and had a history of rheumatoid arthritis, osteoporosis, and dyslipidemia.

The patient had difficulty finding words and speaking from approximately 68 years of age and visited our hospital at 69 years of age. According to her son, she had spoken slowly, though she used to speak quickly. He also pointed out that she might have hearing problems regarding the comprehension of words. At the age of 70 years, the patient complained that she had difficulty understanding words and could not speak smoothly when words included voiced sounds and double consonants. At 71 years of age, though she stated ‘I can hear sounds but cannot hear them as words’ and had difficulty speaking, she could work at the cash register in the restaurant and deal with tax accountants. The patient could perform most of her activities of daily living (ADLs) without assistance at the age of 72 years; however, she became unable to engage in conversation and thus communicated in writing.

Although the patient purchased hearing aids at the age of 72 years, they were ineffective. She could hear sounds but could not understand words. She was still engaged in work at the age of 74 years, although she had impairments in understanding and expressing spoken language. Her consciousness was clear, and she was neurologically normal, except for moderate hearing loss.

Methods

From the age of 69 years, the patient underwent a standard protocol of neurological and neuropsychological examinations, language and auditory assessments, and neuroimaging. Ethics review and approval were not required for this study in accordance with the local legislation and institutional requirements. Written informed consent for participation was obtained from both the patient and her family members.

Neuropsychological and Language Assessments

To evaluate her cognitive abilities, the Mini-Mental State Examination (MMSE), the Alzheimer’s Disease Assessment Scale (ADAS), Wechsler Memory Scale-Revised (WMS-R), Rey-Osterrieth Complex Figure Test (ROCFT), Wechsler Adult Intelligent Scale-IV(WAIS-IV), and Frontal Assessment Battery (FAB) were undergone. As language assessments, we administered the Western Aphasia Battery (WAB) Token Test to investigate the features of her language processing.

Auditory Assessments

For examining the patient’s auditory abilities, auditory brainstem response (ABR), and pure-tone audiometry, the speech reception score monosyllabic word list was administered at the age of 73 years.

For the environmental sounds, 18 types of sound sources were used, including police car sirens, railroad crossings, bird calls, insect sounds, musical instruments, and songs (seven elderly healthy controls (average age: 74.7 years) scored an average of 17.1 of the 18 sound stimuli). As there were no validated environmental sound tests, we used familiar sounds by reference to previous studies [12,13].

At the age of 74 years, the patient was also examined for pure-tone audiometry threshold values. To assess the temporal auditory acuity, click counting and fusion tests were performed [15]. In the click-counting test, the patient was asked how many clicks she heard per second. In the click fusion test, the patient was presented with two clicks at relatively short intervals and asked whether she had heard one or two clicks.

Neuroimaging Assessments

The patient underwent 3-Tesla volumetric head magnetic resonance imaging (MRI). The three-dimensional T1-weighted images taken at age 72 were analyzed using VSRAD® (Voxel-based specific Regional Analysis system for Alzheimer’s Disease) to assess the degree of atrophy of the parahippocampal gyrus. N-isopropyl (^123^I)-p-iodoamphetamine single-photon emission computed tomography (SPECT) once every one or two years. She also underwent (^123^I) 2β-carbomethoxy-3β-(4-iodophenyl)-N-(3-fluoropropyl) nortropane SPECT (DaTscan®, GE Healthcare, Chicago, IL) to investigate the possibility of diseases such as corticobasal degeneration and progressive supranuclear palsy that have extrapyramidal abnormalities.

Results

Neuropsychological Assessments

Throughout the course of treatment, the patient was well-mannered, cooperative in examinations, and aware of her disease. The neuropsychological test results are presented in Table 1.

**Table 1:** Performance on the neuropsychological and language assessments. MMSE = Mini-Mental State Examination, ADAS = Alzheimer’s Disease Assessment Scale, WMS-R = Wechsler Memory Scale-Revised, ROCFT = Rey-Osterrieth Complex Figure Test, WAIS-IV = Wechsler Adult Intelligence Scale Fourth edition, FAB = Frontal Assessment Battery, WAB = Western Aphasia Battery, AQ = Aphasia Quotient, SD = Standard Deviation, Y/O = Years Old ( ): the scores when instructions were presented by writing.

		69 y/o	70 y/o	72 y/o	73 y/o	Normative data mean (SD) and cut-off score
MMSE		29	26	10 (25–28)	2 (22)	23/24
ADAS		-	5.3	26	21.6 (6.6)	9/10
WMS-R	Verbal memory	-	87	50	(50)	100 (15.0)
	Visual memory	-	116	102	(100)	100 (15.0)
	Attention	-	79	<50	(<50)	100 (15.0)
	Delayed recall	-	102	79	(81)	100 (15.0)
ROCFT	(Copy/delay: 30 min. later)	-	34/23	-	-	28/13 (3/4)
WAIS-IV	FIQ	-	100	-	-	100 (15.0)
	Verbal comprehension	-	79	-	-	100 (15.0)
	Perceptual reasoning	-	116	97	-	100 (15.0)
	Working memory	-	79	-	-	100 (15.0)
	Processing speed	-	127	82	-	100 (15.0)
FAB		-	14	-	-	11/12
WAB	AQ	89.5	87.4	12.2	9 (20.6)	97.3 (3.0)
	Auditory comprehension	8.9	9.4	1.7	1.1 (6.3)	9.8 (0.1)
	Repetition	8.8	7.6	0.6	0.3	9.9 (0.3)
	Naming	9.1	8.7	0.8	0.1 (0.7)	9.5 (0.6)
	Reading	9.0	9.6	7.4	5.3	9.5 (0.8)
	Writing	9.6	9.8	4.8	6.1	9.6 (1.0)
Token test		-	158/167	-	-	157/158

The MMSE and ADAS scores were fairly good until 70 years of age. On the WMS-R, visual memory was preserved; however, verbal memory was slightly deteriorated. The delayed recall was favorable. The WAIS-IV also showed a decline in verbal comprehension and working memory but not in perceptual reasoning or processing speed. No apraxia, agnosia, visuospatial cognitive impairment, constructional disorder, or memory impairment were observed, and ADLs were independent. From the age of 72 years, the patient’s auditory comprehension became impaired, and the scores on the MMSE and ADAS significantly decreased; however, the effect of written instruction was maintained considerably, even at the age of 73 years.

Language Assessments

In the language tests shown in Table 1, the scores of the WAB were favorable until approximately the age of 70 years, except for repetition, which was defective in long phrases and sentences, and phonological paraphasia and AOS were recognized but exploratory behavior that rephrased sounds led to correct answers in many cases. At the age of 72 years, tasks requiring spoken language comprehension and expression were almost impossible to perform. The patient exhibited a large dissociation between spoken and written instructions.

Regarding spoken language comprehension, the patient complained of difficulty in understanding words at the age of 70 years; however, no deterioration in spoken language comprehension was observed on tests at this time. Written language comprehension was competent until approximately 70 years of age. Although the written language comprehension of the patient was no longer comprehensive at the age of 72 years, it was superior to that of spoken language; she could communicate in writing and use a smartphone voice-to-text conversion application.

Regarding auditory language expression, the patient’s spontaneous speech was non-fluent and not verbose. At the age of 70 years, she had phonological paraphasia and AOS in spontaneous speech and naming, especially in repetition. Utterance required some effort; the sound was distorted, and the distortion fluctuated. The patient showed frequent rephrasing in repetition, and word-length effects were found for hiragana and katakana (Japanese syllabograms) words. When a word was longer, exploratory behavior was shown, such as, ‘ro-pure-to → do-pure-to → do-pure-to → do-e-puto' (ropeway). In some cases, words usually written in kanji characters (Japanese morphograms) can be repeated, even if they contain a large number of morae. At the age of 72 years, logoclonias began to occur frequently, where the patient repeated one sound in the middle or at the end of words.

The following examples of logoclonia were observed. In Spontaneous speech, the patient spoke in nonsensical utterances, such as ‘kokokototo-rererere’ in response to the question ‘What does this mean?’. In response to ‘What month and day is it today?’, the patient answered ‘hichichichichichi kuukokokokotsutsutsu ninijyuurokokokokokokohihihi’ (26th April; actual pronunciation is ‘shichi-gatsu nijyuu-rokunichi’). She named “pencil” ‘enpitsutsu, enpikukukutete’ (‘enpitsu’ in Japanese pronunciation) and “ball”‘bo-rurururu’ (‘bo-ru’). In repetition, responses such as ‘Sanju sasasa, sanju kyuu’ (33; ‘sanjyu-san’) and ‘Kushi-shi-shi-shi'’ (comb; ‘kushi’) were observed. She recalled in verbal fluency task (animal names), ‘hatototo, inununu, nekokokoko, rakkokoko’ (dove, ‘hato’; dog, ‘inu’; cat, ‘neko’; and sea otter, ‘rakko’), and in word reading, she read ‘orenjijijiji’ (orange; ‘orenji’), ‘banana nanana’ (banana; ‘banana’), ‘momomo, momo, momo’ (peach; ‘momo’). The features of her logoclonia were similar to those of patients with Alzheimer’s disease (AD). The sounds were not distorted different from AOS and their speed was fast and effortless. Even when the patient was instructed to separate each syllable while folding her fingers, the same syllable was frequently repeated rapidly, regardless of the number of times she folded her fingers.

Regarding written language expression, no difficulties were found in spontaneous writing and dictation of kanji characters at the age of 69 years. In writing kana characters or words, phonological paragraphias frequently occurred in loan words and longer words, and the patient often corrected target words repeatedly. At the age of 70 years, her writing presented omitting okurigana, which are hiragana that appear after kanji in Japanese words to indicate the grammatical function, pronunciation, or inflection of the word, parts of words, and postposition particles that are similar to preposition in English.

At the age of 72 years, these became more prominent, and although the patient frequently wrote only kanji words, she could communicate by writing. In response to text questions using handwriting or an application that converts voice to text on a smartphone, the patient frequently wrote kanji words or sentences without postposition particles or endings. However, the patient seemed able to understand the questions to some extent (if I asked her, ‘Are there any problems?’, she wrote, ‘not good at (writing) kana’. In response to, ‘Did you come by yourself?’, she answered, ‘a person comes’.

Auditory Assessments

The patient’s ABR showed V waves up to 60 dB bilaterally, and pure-tone audiometry showed mean hearing of 62.5 dB and 57.5 dB in the right and left ears, respectively (quartile method). The speech reception score monosyllabic word list was 5% and 0% at 100 dB in the right and left ears, respectively. These results confirmed that there was moderate hearing loss; however, a deterioration of speech comprehension ability that could not be explained by hearing loss alone was recognized.

For the environmental sounds, the patient responded to nine types of sounds correctly by writing or forced-choice questions; therefore, writing was considered more advanced than speech recognition. At the age of 74 years, the patient was also examined for pure-tone audiometry threshold values, which were 47.5 dB and 41.3 dB in the right and left ears, respectively. These values did not change throughout the year.

In the click-counting test, the patient was asked how many clicks she heard per second. She counted three and four clicks in the right and left ears, respectively. This was lower than that of healthy individuals, who counted 9-11 clicks per second [16]. In the click fusion test, the patient showed binaural fusion at intervals of 67 ms and 57 ms in the right and left ears, respectively. These intervals were poorer than those in normal controls (normal range: 1-3 ms) [16].

Neuroimaging Assessments

**Figure 1:**
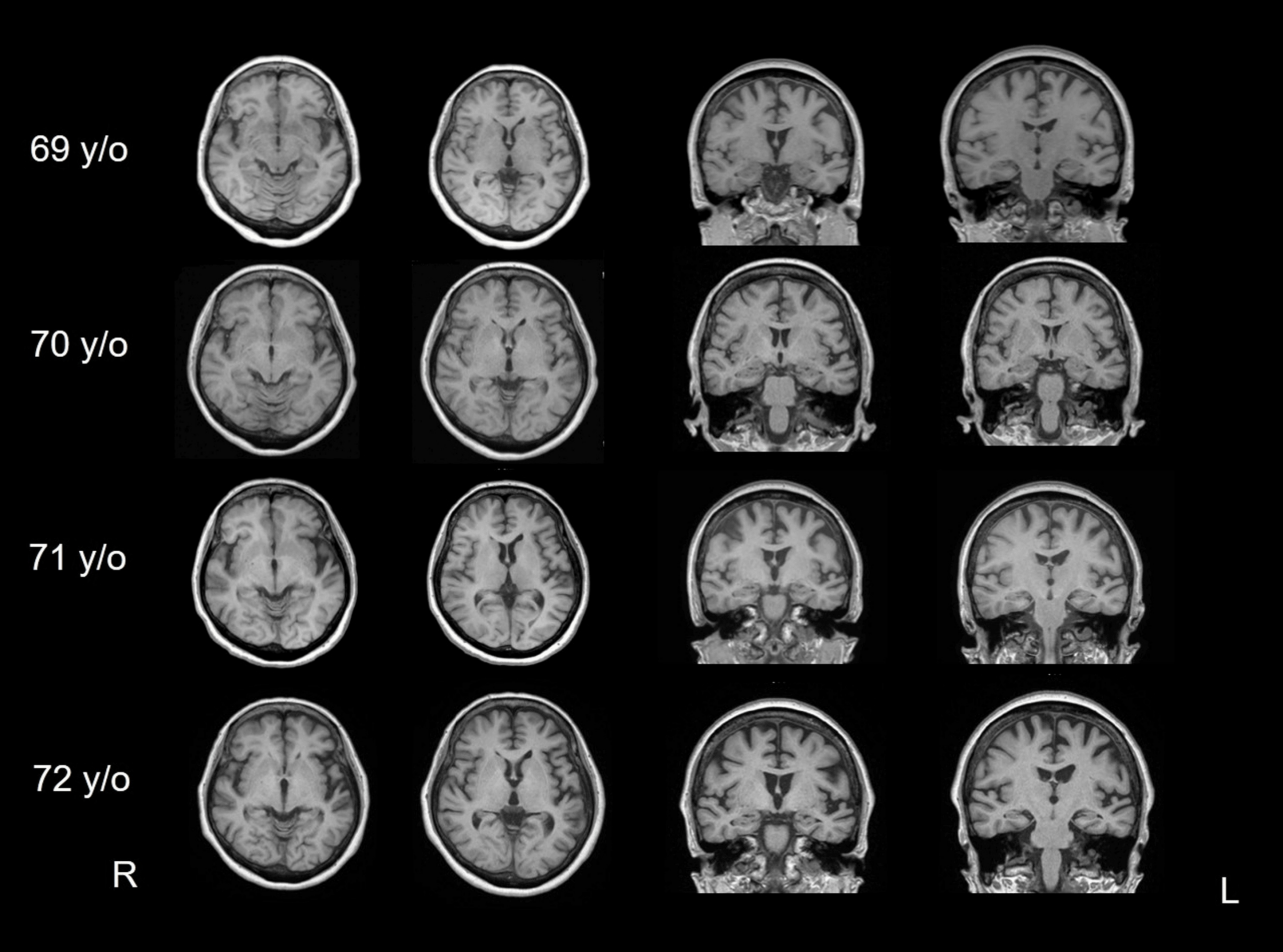
Serial T1 brain magnetic resonance images. L, left; R, right; y/o, years old

MRI revealed dilatation of the left Sylvian fissure and atrophy of the superior temporal gyrus from approximately 71 years of age (Figure 1). The three-dimensional T1-weighted images taken at age 72 were analyzed using VSRAD®. The analysis of the VSRAD® results showed that the degree of atrophy (Z-score) of the medial temporal lobe was 0.8 (<1.0), indicating that there was almost no atrophy.

**Figure 2:**
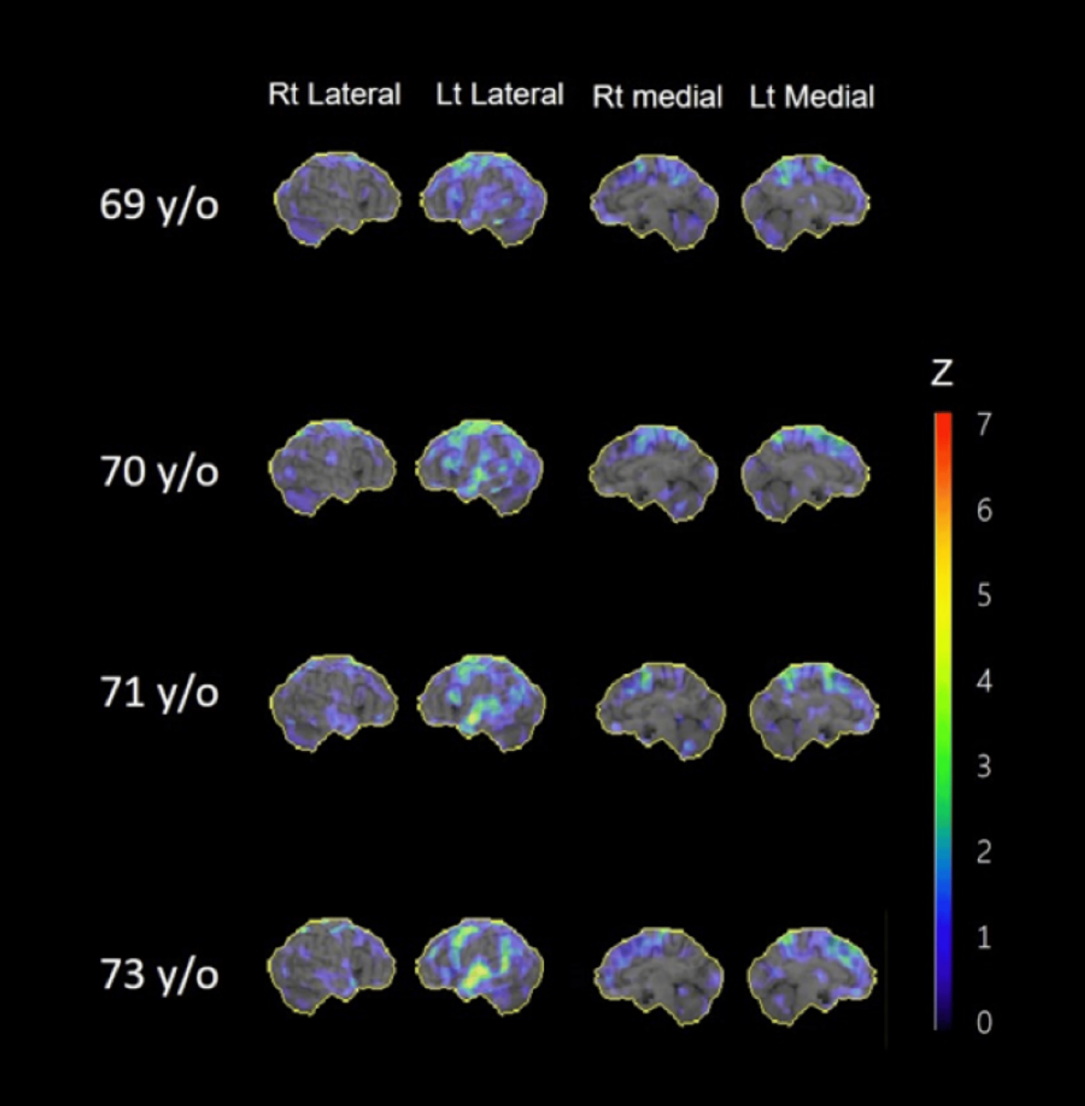
Serial results of single-photon emission computed tomography (three-dimensional stereotactic surface projection decrease). Lt, left; Rt, right; y/o, years old

The three-dimensional stereotactic surface projection Z-score maps normalized to the global brain are shown in Figure 2. Blood flow was reduced in the left hemisphere, particularly in the superior temporal gyrus and lateral frontal lobe.

**Figure 3:**
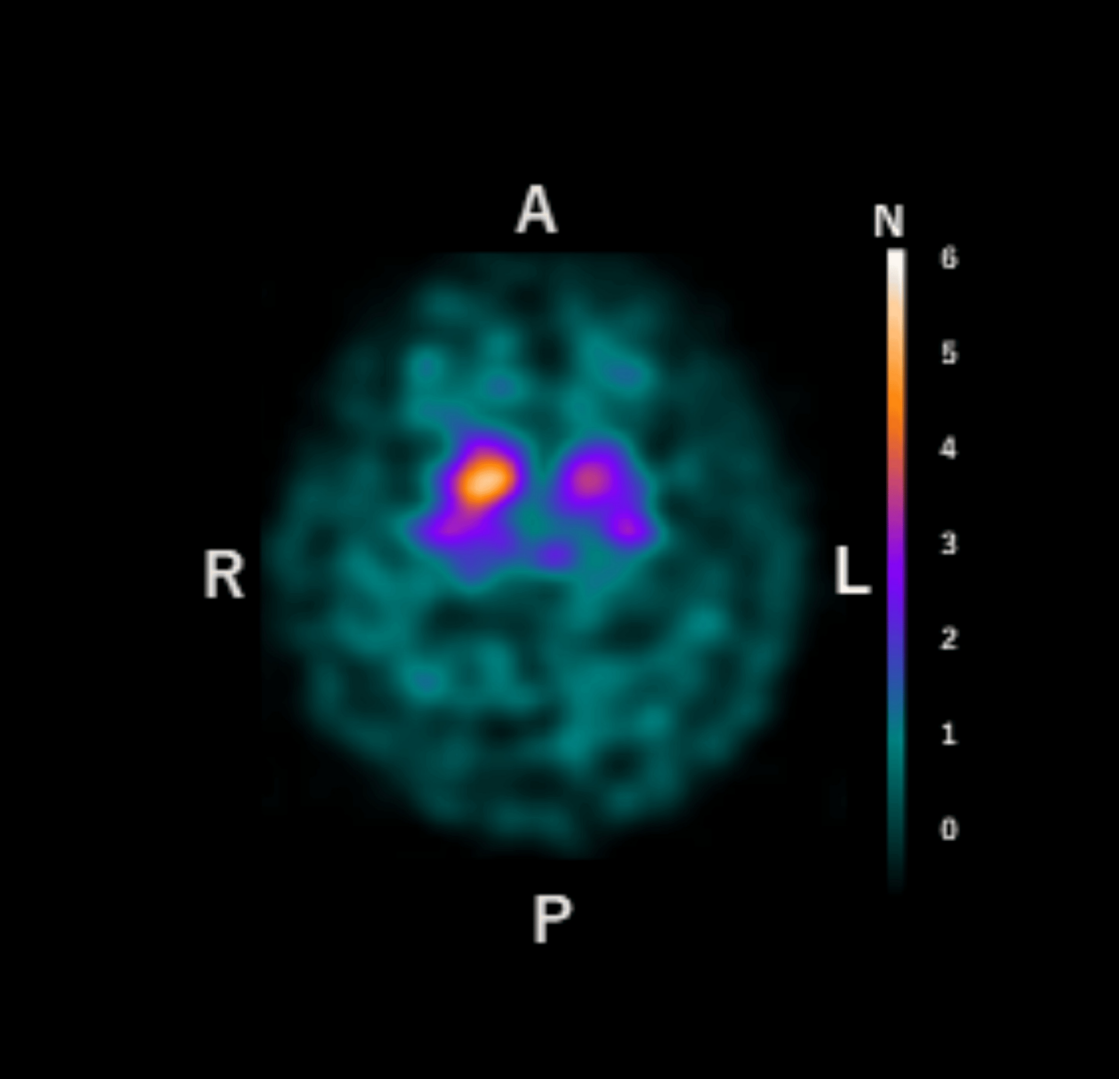
Dopamine active transporter scan. A, anterior; P, posterior; R, right; L, left

The DAT-scan (at the age of 73 years) showed specific binding ratio values of 6.02 (right), 4.29 (left), and 5.15 (average) and an asymmetry index of 33.5%. Decreased uptake in the left striatum was observed (Figure 3).

Cerebrospinal Fluid Analysis

Cerebrospinal fluid examination suggested AD pathology was unlikely: amyloid beta (Aβ) (1-40), 12,359 pg/mL; Aβ (1-42), 1,605 pg/mL; and Aβ40/42, 7.7. The level of tau protein phosphorylation was 57 pg/mL (normal value is <50.0 pg/mL).

## Discussion

A few years after onset, the patient experienced phonological paraphasia and AOS. Although she developed a speech disorder at approximately 70 years of age and complained of difficulty in understanding words, almost no deterioration in auditory comprehension was observed on cognitive function tests. In the early stages of the disease, the patient presented with repetitive disorders; phonological paraphasia and AOS were recognized in foreign words, phrases, and sentences. In writing, some parts of the words and postposition particles were omitted, and only kanji words were arranged. Although her syntactic comprehension was impaired in complex sentences, she had no difficulties with word comprehension or object recognition.

However, at approximately 71 years of age, the patient complained that she could hear sounds but could not understand them as words. The patient lacked full comprehension of written language; however, it was superior compared to her auditory comprehension. Compared to her moderate hearing loss at the age of 73 years, the patient’s speech-hearing ability was markedly reduced, suggesting that she had progressive word deafness. Regarding spoken language expression, logoclonia was observed at the age of 72 years, when she frequently repeated the middle and final sounds of words. During the progression of the disease, deafness and logoclonia became more pronounced. Few reports exist on cases of combined word deafness and logoclonia, and no reports have examined the symptoms longitudinally, including data from VSRAD, DaTscan, and cerebrospinal fluid tests.

Regarding the patient’s speech disorder, the AOS was prominent in the early stages of the disease. AOS is a speech-motor programming disorder characterized by fluctuating articulatory distortions [4], delays in speech speed, and exploratory behavior, frequently accompanied by prosody disturbances and speech difficulties [17]. Additionally, from the age of 72 years, logoclonia, the repetition of one sound in the middle or end of a word, frequently occurred. Logoclonia is defined as the involuntary addition of unaccented syllables, the appearance at the end of words or sentences, and the rapid repetition of the last syllable three or four times or more [18]. Our patient demonstrated these characteristics and had a similar type of logoclonia which has been observed in AD [19-21]. It has also been associated with sensory aphasia owing to cerebral infarction and PPA [10,11,22,23]. Moreover, there are reports of logoclonia in each PPA type (nfvPPA, svPPA, and lvPPA) [18,24,25].

Some studies have reported that logoclonia occurs when the lexical and semantic aspects of language are significantly disturbed [20,25,26]. However, it has been reported that ADLs are unaffected, and the internal language is relatively well preserved [10,11,18,21,23]. A few reports that capture changes in speech disorders over time exist [22]; however, in this case, written language expression was maintained even at the age of 73 years, suggesting that logoclonia occurs at the level of executing utterances as articulation movements or at the level of regulating the motor output of speech [11].

Regarding the impairment of verbal sound cognition, the patient complained of difficulty in understanding words from approximately 70 years of age. From the age of 72 years, this difficulty became evident in the dissociation between written and auditory instructions in the MMSE. The patient showed moderate hearing loss based on the results of pure-tone hearing and ABR tests at the age of 73 years. Compared to her hearing loss, speech sound hearing was markedly degraded. Environmental sounds were also impaired; however, the degree of impairment was considered milder than that of speech sounds. There have been reports of word deafness and auditory agnosia in neurodegenerative diseases [5-7,27-30], suggesting that the auditory comprehension impairment in this case was also primarily owing to progressive word deafness. Several cases of progressive word deafness or auditory agnosia with speech disorders associated with auditory-language disorders have been reported as follows: word deafness accompanied by prosody disorders [7], paragrammatical and stereotypical expressions [31], FAS [9], AOS [5,6,27,29,30], and logoclonia ([22], a progressed case of Ota et al. [9]), and auditory agnosia with logoclonia [10]. In patients with nfvPPA, there are also cases of combinations of word deafness and AOS [12,29]. These reports are summarized in Table 2, in which we included not only the features of language impairments but also behavioral impairment because patients tend to have that kind of impairment as the disease progresses [22]. A PubMed search for the combination of word deafness, logoclonia, and progressive aphasia yielded three results, including two case reports [18,25]. Nakagawa et al. [25] reported a case of AD, which differs from our case. Shimazaki et al. [18] reported a patient with nfvPPA, similar to our case; however, the patient was deaf in his right ear, had moderate hearing loss in his left ear, and was not diagnosed with word deafness. Additionally, they did not clearly describe the relationship between auditory impairment and speech disorders.

**Table 2:** Reported cases of having slowly progressive auditory impairments and difficulties in speaking. FAS = foreign accent syndrome, speaking→auditory: the case showed speaking disorders first and then auditory impairments, auditory→speaking: inverse of speaking→auditory, auditory = speaking: the patient showed speaking disorders and auditory impairments simultaneously

	Speaking disorders	Agrammatism	Behavioural impairment	Relationships between auditory impairments and speaking disorders
Sakurai et al. [31] (case 4)	Stereotypical expression?	paragrammatism	-?	speaking→auditory?
Otsuki et al. [7]	Prosody disorders	-?	-	auditory→speaking
Lee et al. [11]	Logoclonia	paragrammatism?	+	auditory→speaking
Kuramoto et al. [10]	Logoclonia, jargon	-?	-	auditory→speaking
Kaga et al. [6]	AOS	-?	-?	auditory=speaking
Iizuka et al. [5]	AOS	-?	+	auditory→speaking
Gibbons et al. [27]	AOS	+?	+	auditory→speaking
Ota et al. [9], Matsuda [22]	FAS→logoclonia	+?	+	auditory→speaking
Sato et al. [29]	AOS	+(a little)	+	auditory=speaking
Utianski et al. [30]	Non-verbal oral apraxia→AOS	+	+	speaking→auditory
Watanabe et al. [12]	AOS	+	-?	auditory=speaking?
Shimazaki et al. [18]	AOS, logoclonia	+	-(euphoric)	speaking→auditory?

Based on the course of the disease in our case, we assume that there are two possibilities for the relationship between speech disorders and word deafness. First, mild word deafness may be associated with speech disorders at an early stage. There have been many reports where speech sound recognition disorders become noticeable first, followed by speech disorders [5-7,9-12,27,29]. Ota et al. [9] suggested that disturbances in neural substrates associated with word deafness may affect speech feedback, resulting in speech disorders. In our case, word deafness may have caused speech disorders, although it was not noticeable in the language tests in the early stages. The second possibility is that deafness and logoclonia became comorbid in our case as the disease progressed after the onset of AOS. In the early stages of the disease, the impairment of the patient’s auditory comprehension was not prominent on some neuropsychological tests. There have been reports of cases where speech disturbances were identified first. There was one patient with slowly progressive fluent aphasia and speech difficulty, followed by word deafness (case 4 in Sakurai et al. [31]). Additionally, a case showed a transition from unclassifiable PPA to agrammatic PPA, which was preceded by AOS, followed by word deafness [30]. Mild deafness or hearing impairment may also be present in such cases. It has been reported that nfvPPA is associated with hearing impairment when compared with AD and healthy subjects, as well as with svPPA and lvPPA [32-34]. These studies indicated that impairments in auditory processing may have been the cause of speech impairments or that both impairments may have been related manifestations. Hardy et al. [34] noted that the results of pure-tone audiometry are frequently impaired in nfvPPA. As the case reported by Watanabe et al. [12] also had mild hearing loss, the moderate hearing loss in our case may also be associated with speech disorder. It has been suggested that the time of appearance of word deafness and speech disorders, and the relationship between the two, differ depending on the timing of the examination and the spread of the lesion.

From the age of 71 years, dilatation of the left Sylvian fissure and atrophy of the superior temporal gyrus were conspicuous in our patient, and single-photon emission computed tomography (SPECT) revealed marked hypoperfusion in the left hemisphere, especially in the superior temporal gyrus and lateral frontal lobe. At approximately this time, the patient complained of difficulty in understanding words, and her speech comprehension was markedly degraded. Speech sounds are input from the bilateral primary auditory cortices to the left secondary auditory cortex (Wernicke’s area). The impairment of this pathway results in pure word deafness [10]. It has been suggested that the lesion is located in the cortical and subcortical temporal lobe [35]. The marked decrease in blood flow in the superior temporal gyrus observed in our case was thought to be consistent with the lesion responsible for word deafness. Concerning speech disorders, the emergence of logoclonia requires neurological disorders centered in the frontal lobe and connecting the basal ganglia, supplementary motor area, or limbic system, and the involvement of extrapyramidal syndrome is assumed [20]. In our case, MRI and cerebral blood flow showed disturbances in the frontal lobe and supplementary motor cortex, and DaTscan results supported the appearance of extrapyramidal symptoms. As a pathological entity, no evidence of AD was found in the cerebrospinal fluid examination, and decreased uptake was observed in the DaTscan, suggesting the possibility of disease within the frontotemporal lobar degeneration area, such as corticobasal degeneration, unlike AD pathology. Previous reports have shown behavioral abnormalities owing to frontal lobe signs over time in patients with progressive word deafness [5,9,11,22,27,29-31]. However, in this case, no behavioral problems were observed at the age of 73 years, and follow-up was necessary.

The limitations of this study included the absence of auditory tests in the early stages of the disease and the lack of a clear temporal course of the relationship between word deafness and speech impairment. During the rapid progression of speech sound cognition and speech impairment at approximately 71 years of age, we were unable to adequately examine the patient’s symptoms. Additionally, impairments were found in environmental sounds, although they were milder than speech sound cognition.

From the course of the disease in our case, it is highly possible that word deafness caused the dissociation between spoken and written comprehension. Speech disorders, such as phonological paraphasia, AOS, and logoclonia, significantly influence the dissociation between spoken and written expressions. Word deafness may be a significant cause of age-related communication disorders; however, it may have been overlooked simply as presbycusis. The significance of being aware of progressive word deafness is that it enables measures that are different from those for hearing loss, such as the fact that hearing aids are not useful as in the case of hearing loss and that comprehension can frequently be improved by dividing each syllable into separate words and speaking slowly. Furthermore, speech disorders can change throughout their progression and transition from AOS or FAS to logoclonia. We believe that longitudinal studies of the relationship between speech disorders and word deafness are significant in understanding the mechanism by which the two co-occur.

Based on the examination of our patient and previous case studies, among degenerative diseases presenting with progressive communication disorders as a different entity from PPA, one group may show auditory processing disorders, such as word deafness and auditory agnosia, and speech movement disorders, such as prosody disorders, AOS, FAS, and logoclonia. Although patients with this syndrome may experience agrammatism, inner language disorders are generally not as severe. Disorders in areas closer to the primary area, such as the motor and auditory areas, as well as cortical and subcortical disorders, are involved in this syndrome.

## Conclusions

From the course of the disease in our case, it is suggested that word deafness caused the dissociation between spoken and written comprehension and speech disorders such as logoclonia largely affected the dissociation between spoken and written expressions. We believe that this case provides new insights into the clinical condition of auditory processing and speech movement disorders.
